# Arthritis glove provision in rheumatoid arthritis and hand osteoarthritis: A survey of United Kingdom rheumatology occupational therapists

**DOI:** 10.1177/17589983211060620

**Published:** 2022-01-05

**Authors:** Alison Hammond, Yeliz Prior

**Affiliations:** Centre for Health Sciences Research, 7046 University of Salford, Salford, UK

**Keywords:** rheumatoid arthritis, hand therapy, arthritis gloves, orthoses, occupational therapy

## Abstract

**Introduction:**

Hand pain and function limitations are common in rheumatoid arthritis (RA) and hand osteoarthritis (HOA). Provision of arthritis (compression) gloves to relieve hand symptoms is increasing in occupational therapy. Research evaluating arthritis gloves dates to the 1990s, focussing on night-wear of full-length finger gloves in RA. This survey examined glove provision in contemporary clinical practice in the United Kingdom.

**Methods:**

A survey of arthritis glove provision in RA was conducted with Royal College of Occupational Therapists Rheumatology Specialist Section members. A more detailed survey about glove provision in RA and HOA was conducted with rheumatology occupational therapists in North-West England.

**Results:**

Response rates were good, with 60 (73%) therapists responding to the national and 24 (69%) to the regional surveys. Most therapists provided open-finger gloves (commonly Isotoner^TM^) to about a third of their RA and HOA patients, and to those with any arthritic condition causing significant hand pain and/or swelling. Day-wear was as common as night-wear, and patients were advised to wear these ‘as and when’ for hand symptom relief and support for hand function. They were advised not to wear gloves continually in the day, and regularly perform hand exercises and monitor for potential adverse effects, for example, skin discolouration. Therapists commonly provide replacement gloves as these are often used long-term.

**Conclusion:**

Prescription of arthritis gloves has changed considerably in the last 30 years, with open-finger gloves provided to a wider range of people with arthritis, for a broader range of clinical reasons.

## Introduction

For over 40 years, arthritis gloves (also called compression or therapy gloves) have been prescribed to people with rheumatoid arthritis (RA) and hand osteoarthritis (HOA) in the United Kingdom (UK), North America and Europe.^1–5^ Anecdotally, they are also prescribed to people with other rheumatic and musculoskeletal conditions and in other countries. They are also sold commercially to people with arthritis worldwide.^6^ Both RA and HOA cause hand pain, joint swelling, stiffness and reduce hand function.^7–9^ Arthritis gloves are prescribed to help reduce these hand symptoms^1–5^ and to provide light support: during activity to improve hand function; and at night to also provide comfort and aid sleep.^10,11^

Most designs of arthritis gloves include elastane (i.e. Lycra or Spandex) within the fabric to apply external compression. For example, the Isotoner^TM^ glove (80% nylon, 20% elastane) provides 23–32 mmHg pressure^12^ (Supplementary File 1: Figure 1: Figure 1). Other gloves exert less compression, for example, the Jobskin® classic oedema glove (89% nylon, 11% elastane), exerting 15–25 mmHg pressure, as these contain less elastane^13^ (Supplementary File 1: Figure 2). Some glove designs include neoprene, for example, the Thermoskin® glove (57% neoprene, 34% polyester, 9% nylon) to apply ‘a light but firm compression’, although the amount is not stated.^14^ The mechanism of action of arthritis gloves is thought to be that external compression helps remove articular and periarticular swelling. As with any glove, glove fabric can also increase warmth in the hands. Pressure and warmth can therefore reduce hand symptoms.^4,5^ Glove elastane or neoprene content and closeness of glove fit influence the amount of pressure applied.

There were several small (i.e. *n* = 8–27), randomised crossover trials in the 1970s to 1990s evaluating the effects of night-wear of closed (i.e. full-length) finger arthritis gloves in RA,^1–5^ one of which also evaluated gloves in HOA.^2^ Two case studies describe day-time glove wear in RA.^10,11^ Two reviews have been published. A narrative review concluded there is evidence that arthritis gloves for RA lead to hand symptom improvement, that is, in nocturnal hand pain, stiffness and swelling, but do not improve hand function. However, studies were included irrespective of design and methodological quality.^15^ In contrast, a systematic review, identified four randomised trials comparing arthritis gloves with placebo gloves not applying any pressure.^16^ Three trials had moderate risk ^1,2,4^ and one high risk^3^ of bias. In the three trials with moderate risk of bias, all three reported significant reductions in proximal interphalangeal joint swelling (by 0.07–1.1 mm.) in RA.^1,2,4^ Trials were conflicting and therefore inconclusive about effects on: nocturnal hand pain (with one trial reporting improvement^1^ and two no significant improvement^2,4^); and stiffness (with one trial reporting improvement,^1^ one no significant differences^2^ and the third similar improvements in both arthritis and placebo gloves^4^ ). In those trials measuring the following, no benefits were identified in hand swelling,^1^ range of motion,^2^ dexterity,^1,2^ grip^1,2,4^ and pinch^1^ strength. No differences in HOA were identified.^2^ One trial reported both arthritis and placebo gloves improved palmar skin temperature by 1°C.^2^

Although arthritis gloves have been provided clinically for many decades, no articles could be identified within the last 30 years describing clinical practice about arthritis glove provision. The aim of this study was to investigate rheumatology occupational therapists’ clinical practice in arthritis glove provision in the UK National Health Service (NHS), with a focus on RA and HOA. The information obtained would then be used in the planning process for a randomised controlled trial to evaluate arthritis gloves.

## Methods

### Study design

The study was a cross-sectional survey. There were two stages: a short online national survey and a more detailed survey conducted in one region of England. Prior to starting the study, ethical approval was obtained from the School of Health and Society Ethics Panel, University of Salford. Therapists were provided with invitation e-mails or letters and participant information sheets. Survey completion was considered indicative of consent.

### Participants and data collection procedures

During questionnaire development, rheumatology occupational therapists indicated that they provided arthritis gloves within their services, rather than rheumatology physiotherapists. At the time there was no specialist rheumatology physiotherapist network to distribute the survey to. Accordingly, we contacted rheumatology occupational therapists only to complete the surveys. An invitation to the national survey was distributed by the Royal College of Occupational Therapists Rheumatology Specialist Section to their members (*n* = 82), who could anonymously complete an online questionnaire (with a paper option available, if preferred). The regional survey was conducted with members of the North-West England Rheumatology Occupational Therapists group (i.e. Greater Manchester, Merseyside, Cheshire, Lancashire, and Cumbria; *n* = 35), with invitations distributed by email, using a paper questionnaire. Respondents could choose whether to add their contact details or not. Provision of contact details allowed for follow-up of missing data. The North-West group is not limited to Specialist Section members. Regional group members could also respond to the national survey if Section members, although were informed separately that they did not need to. One reminder was sent in both surveys.

### Questionnaire content

There was no pre-existing validated or published questionnaire about arthritis glove provision. Therefore. an original questionnaire was jointly designed with members of the North-West Rheumatology Occupational Therapists Group, who helped generate and agree items based on literature review and discussions about current practice, to provide face validity. Discussion identified that gloves are not only used in RA but also increasingly in HOA, as well as other hand conditions. The regional survey included 12 questions on glove provision in RA, plus options about HOA, and 22 additional questions to obtain more detailed information about arthritis glove clinical practice. We used the 12 questions on glove provision in RA only in the national online survey (Supplementary File 2) for brevity, to encourage a good response rate and reduce responder burden.

Both surveys included: socio-demographic data: NHS job band; settings work in; years of Rheumatology experience; and if therapists provided gloves or not (and if not, why not. Questionnaire completion then ended). For those providing arthritis gloves, both surveys included items about estimated numbers of early and established RA patients/month receiving gloves/month. (For the regional survey only, this was also asked about HOA, as well as estimated percentage of RA and HOA patients prescribed gloves). Both surveys also included what type of gloves are provided; wear regimens; replacement glove provision and if patients needed to pay for these. The North-West England region survey also asked about:

• Clinical decision-making: therapists’ rationale for and explanations to patients about glove provision; aims of and factors prompting glove provision; and glove choices.
• Glove provision: assessment for; wear regimens; instructions; contraindications; glove review and evaluation; and patient feedback on benefits and drawbacks of arthritis glove wear.

### Data analysis

Data was descriptively analysed using: numbers and percentages; means and standard deviations (interval data); and medians and interquartile ranges (ordinal data) using SPSS v26.^17^ Qualitative responses were content analysed, as responses were generally brief. Data were read, coded and categorised by two researchers and presented either as themes or as numbers of respondents stating key categories, as applicable.^18^ Regional survey data on numbers of gloves provided per month in RA and HOA and time taken to provide gloves was used to estimate average annual costs of glove provision for rheumatology occupational therapy departments. NHS arthritis glove costs were identified from rheumatology therapists (2020 prices). Staff costs were identified using published NHS staff cost data for 2020.^19^

## Results

### Participants

In the national survey, 60/82 (73%) responded. In the North-West region survey, 21/35 questionnaires were returned, with three being completed by two therapists each from the same departments, that is, a 69% (24/35) response rate from 21 rheumatology occupational therapy departments. (Socio-demographic information was only provided for one therapist at each of these three departments). Socio-demographic characteristics are shown in Table 1.

**Table 1.** Demographic characteristics of arthritis glove survey respondents.

	National survey (*n* = 60)	Regional survey (*n* = 21)
*National health service job band*		
Band 5	2	0
Band 6	30	4
Band 7	23	16
Band 8A and above	5	1
*Work settings (n)*		
District general hospitals	28	10
Teaching hospitals	8	4
Community hospitals	8	2
Other	16	5
*Years’ experience in rheumatology*		
less than 1 year	1	0
1–3 years	4	0
4–5 years	7	4
6–10 years	12	3
More than 10 years	36	14

### Glove provision: national and regional survey findings

Most therapists provided arthritis gloves: nationally, 58/60 (97%) and in the North-West region, in 18/21 (86%) departments. (One response was incomplete meaning most regional survey results are from 17 departments. This respondent was contacted about missing items but no reply received). Reasons for not providing gloves were: national (*n* = 2): no budget for gloves (*n* = 1); and consider resting splints a better option (*n* = 1); and regional (*n* = 3): no clinical experience of glove provision (*n* = 2), no budget for gloves (*n* = 1).

#### Hand conditions

Of those providing gloves, in both surveys most (90–100%) therapists provide gloves in both early and established RA. For HOA, a third nationally and most in the North-West region provide gloves (Table 2). Additionally, in the national survey an open question asked what other conditions gloves are provided in. These were: any arthritic condition causing significant hand joint pain and/or swelling (*n* = 24); psoriatic arthritis (*n* = 9); hypermobility (*n* = 6); fibromyalgia (*n* = 4); carpal tunnel syndrome (*n* = 2); Raynaud’s disease (*n* = 2); gout (*n* = 1); lupus (*n* = 1), and de Quervain’s (*n* = 1). Based on respondents’ estimates, in the regional survey therapists provide gloves to around a third of patients with RA or HOA that they treat, although this varied considerably between departments from: 5–90% in early RA; 10–60% in established RA; and 5–70% in HOA (Table 2). Their use in early RA was often short-term (e.g. up to 6 months) to assist improving hand symptoms and function whilst waiting for disease modifying anti-rheumatic drugs (DMARDs) to become effective.

**Table 2.** Arthritis glove service provision in rheumatoid arthritis and hand osteoarthritis.

	National survey (*n* = 58)	Regional survey (*n* = 18)
Provide compression gloves: yes (*n*: %)		
Early RA	52 (90%)	18 (100%)
Short-term use prior to DMARDS becoming effective	45 (76%)	16 (89%)
Early RA when stable on DMARDs	37 (64%)	17 (94%)
Established RA	49 (84%)	17 (94%)
HOA	22 (38%)	17 (94%)
RA		(*n* = 17**)
No. patients prescribed gloves/month: Mean (SD)	10.55 (8.48)	13.40 (9.42)
Estimated % early RA patients provided with gloves*	—	37 (SD 30)
Estimated % established RA patients provided with gloves*	—	30 (SD 19)
HOA		
No. Patients prescribed gloves/month:Mean (SD)	—	6.88 (6.15)
Estimated % HOA patients provided with gloves*	—	32 (SD 22)

Key: RA = rheumatoid arthritis; HOA = hand osteoarthritis; DMARDs = disease modifying anti-rheumatic drugs; SD = standard deviation.

* Based on respondents’ estimates.

** one respondent did not answer these questions.

#### Glove types and designs

Two-thirds of respondents could provide only one type of arthritis glove, with Isotoner™ gloves tending to be more common. For those providing oedema gloves, one brand was available (either Jobskin, Patterson Medical, Promedics or Sammons Preston). Around a third of respondents could provide both Isotoner and oedema gloves (national: 18/58 (31%); regional: 7/17 (41%). Nationally, eight (14%) also provided Thermoskin® gloves. A few manufactured their own bespoke gloves when necessary (national *n* = 2; regional *n* = 5). Most therapists provided gloves with an open-finger design (i.e. finger ends exposed to allow easier hand function). A third could also provide closed-finger gloves. It was unusual to only stock closed-finger gloves (national: *n* = 4 (7%) (Table 3).

**Table 3.** Arthritis provision in rheumatoid arthritis and hand osteoarthritis.

	National survey (*n* = 58)	Regional survey (*n* = 17*)
*Glove type prescribed n (%)*		
Isotoner™	39 (67%)	15 (88%)
Oedema	36 (62%)	10 (59%)
Thermoskin	8 (14%)	—
*Glove design n (%)*		
Open finger	50 (86%)	17 (100%)
Closed finger	23 (40%)	5 (29%)
*Wear regimens n (%)*		
Day-time	40 (67%)	17 (100%)
Night-time	46 (77%)	17 (100%)
During a flare-up (in RA only)	43 (72%)	15 (88%)
*Replacement gloves provided n (%)*	42 (73%)	14 (82%)
Payment required for spare or replacement gloves	1 (2%)	1 (6%)
Provide contact details for self-purchasing spare or replacement gloves	45 (78%)	11 (65%)

Key: RA = rheumatoid arthritis.

* one respondent did not answer these questions;

#### Glove provision and replacement

Most indicated they provided one pair of gloves at a time (or a single glove if a unilateral hand problem). Most respondents could provide replacement gloves when these wore out. However, in the comments section (national survey), 10 stated that either they could not replace gloves, or soon would be unable to, due to orthotics budget restrictions, or they were limited to providing one or two pairs of replacement gloves only. Very few departments asked patients to pay for additional gloves, with most recommending where patients could purchase spare or replacement gloves of the same type and size instead to ensure good quality, well-fitting gloves were purchased (Table 3).

### Glove provision: North-West Region survey findings

Therapists considered factors influencing their decision to provide arthritis gloves as a treatment option were: attending courses and other therapists’ recommendations (*n* = 15); positive feedback from patients about hand symptom and function improvements during glove wear (*n* = 9); the available research evidence (*n* = 6); and audit results (*n* = 2).

### Mechanisms of glove action and explanations to patients

Most considered that compression was the mechanism of action of arthritis gloves, by reducing oedema, swelling and/or inflammation (*n* = 14). Two did not respond and one stated that ‘there is a poor understanding of the physiological processes involved’. Therapists further considered that: reduced swelling led to pain reduction (*n* = 7); compression provides support to joints (*n* = 6) and can help reduce stiffness (*n* = 2); and these effects improve hand function (*n* = 4), which may also be aided through the proprioceptive feedback from glove wear (*n* = 2). Warmth was also considered to provide pain relief and relax muscles (*n* = 3).

Most explained the rationale for providing gloves to the patient (*n* = 15). Themes reflected the therapists understanding (therapists’ Personal Identification Numbers are given in brackets):

- *Gloves help reduce swelling, pain and stiffness*: for example, ‘..specifically designed to help reduce swelling and relieve pain in the hand… Wearing the glove provides gentle compression and massages the hand helping to reduce swelling’ (P03).
- *Support and comfort*: for example, ‘gloves provide gentle, even support to the hand, preventing muscle strain and fatigue’ (P04).
- *Improve function*: for example, ‘The right amount of compression to help reduce/control swelling…to help improve function’ (P13).
- *Warmth can help*: for example, ‘Increased surface temperature helps with stiffness’ (P06).

### Clinical decision-making about glove provision

Most (*n* = 16) stated it was the therapist’s clinical decision to provide arthritis gloves, and a third (*n* = 5) also received glove referrals from rheumatology consultants. The most important aims of glove provision in RA and HOA were: to reduce hand pain (day and/or night), general hand swelling and stiffness; and improve hand function (Table 4). Factors influencing therapists’ decision to provide gloves to a patient with RA or HOA were: high hand pain levels in the day and/or night, hand joint swelling; the need for hand support in the day; to reduce sleep disturbance from hand pain; and to reduce early morning stiffness. Patient preferences were also taken into consideration, such as a patient may prefer to wear arthritis gloves as easier to tolerate than firmer splints in the day or night; and being more acceptable to patients with early RA (Supplementary file 3). Reasons for choosing to provide Isotoner, oedema or bespoke gloves, and modifications made to gloves are summarised in Supplementary File 4.

**Table 4.** Importance of glove provision aims in early and established rheumatoid arthritis and in hand osteoarthritis (median; interquartile range: North-West region survey (*n* = 17).

Aims	Early RA (<2 years)	Established RA (>2 years)	HOA
Reduce pain during the day	5 (4–5)	5 (4–5)	5 (5–5)
Reduce pain at night	5 (4–5)	5 (4–5)	5 (4–5)
Improve hand function during day	5 (4–5)	5 (4–5)	5 (4–5)
Reduce general hand swelling	5 (4–5)	5 (4–5)	4 (2–5)
Reduce morning stiffness	4 (3–5)	4.5 (3.25–5)	3 (2–4)
Rest/support joint to reduce inflammation	3 (3–4.5)	3 (3–4.5)	3 (2–4)
Increase joint stability	2 (1.5–4)	2 (1.5–4)	3 (1-4)
Minimise risk of deformity	1 (0–2)	1 (0–3)	0 (0–2.75)
Correctly position joints with developing deformity	1 (0–1.75)	1 (0–2.5)	1 (0–1)
Minimise joint contractures	0.5 (0–1)	0.5 (0–1.75)	0 (0–3)

Key: RA = rheumatoid arthritis; HOA = hand osteoarthritis.

Scale: 0 = not important/relevant; 1 = low importance; 5 = high importance.

### Glove provision

Half of therapists used standardised assessments to decide whether to provide gloves (Table 5), either combined with or instead by: questioning to elicit symptoms (*n* = 6); observation (*n* = 6); and discussion (*n* = 5). Contraindications to glove provision are listed in Table 5. Therapists recommended glove wear for RA and HOA during the day, night and/or in a flare-up (for RA) (Table 3), with recommendations for duration of wear being individualised:

• ‘*As and when’ (day and/or night)*: for example, ‘use on activity as needed’ (P01); ‘when hands/joints are swollen, painful or stiff (P03)’; ‘when they feel the need (P14)’; and ‘when they find them most helpful (P18)’.
• *Overnight*: ‘to ease pain and swelling’ (05); and if have ‘hand stiffness/swelling on waking’ (P04).

**Table 5.** Arthritis glove assessment, contraindications, review and evaluation: North-West regional survey (*n* = 17).

	*n* =
Initial assessment	
Standardised assessments	9
Pain visual analogue scale	8
Swelling (tape measure; oedema gauge; ring sizer)	7
Grip (dynamometer)	3
Movement (goniometer)	3
Patient reported outcome measures (Disabilities Arm Shoulder Hand questionnaire or Canadian Occupational Performance Measure)	3
Contraindications	
Skin conditions/allergies potentially exacerbated by glove wear	8
Difficulty don/doff gloves (e.g. por pinch strength, finger deformity)	8
Neurological symptoms (e.g. pins and needles; carpal tunnel syndrome; sensory impairment due to diabetes)	7
Hand circulation problems	7
Difficulty understanding correct glove use	3
Glove review	
Face-to-face review: 4 (SD 2.5 weeks) after provision	12
Telephone review: 4 (SD 2.4) weeks after provision	4
Patient to contact if problems	1
Glove evaluation	
No routine evaluation (ad hoc at annual review or later appointment)	11
Verbal feedback	9
Frequency of glove use	6
Standardised assessments (as above)	3

Key: SD = standard deviation.

Therapists took on average 25 (SD 20) minutes to assess for, fit, and explain instructions and precautions about gloves. Additional advice given to support patient safety is summarised in Table 6. Therapists routinely taught patients hand exercises, for on average 12 (SD 6) min. Glove reviews were usually conducted face-to face, but also by telephone, around 4 weeks after provision (Table 5). Face-to-face glove reviews took on average 15 (SD 11) min or telephone reviews 7.6 (SD 2) min. The average time taken overall to provide arthritis gloves, teach hand exercises and review gloves in person was 52.4 (SD 20.5) min.

**Table 6.** Additional advice provided by therapists to support patient safety. North-West region survey (*n* = 17).

Advice to support patient safety
*Not to wear gloves continually in the day* ‘Discourage constant use’ (P05); ‘not to wear for longer than a few hours at a time’ (P14); ‘to remove regularly for hand hygiene’ (P15)
*Build up tolerance* ‘Trial for one hour initially’ (P21); ‘try in the evening building up tolerance to use before trying overnight’ (P01)
*Regularly remove, check for specific adverse effects* ‘Check skin for red areas’ (P02); ‘observe if causing any circulation or sensory problems…remove and discontinue wear if any problems and contact OT’ (P03); ‘check if any change in colour of fingertips or pressure areas’ (P25)
*Do hand exercises when removed (in morning after night-wear; and during the day)* ‘A simple hand exercise regimen on removal’ (P05) Most taught patients hand exercises routinely when providing arthritis gloves (range of motion *n* = 16; strength *n* = 7)
*Glove care (following manufacturer’s instructions)* ‘Regularly handwash in lukewarm soapy water and air dry’ (P15)
*Driving* Over half specifically recommended patients not to wear arthritis gloves when driving (*n* = 10); and the remainder (*n* = 7) that they could but with care Two-thirds (*n* = 12) recommend patients inform the Driver and Vehicle Licensing Authority that they wore arthritis gloves when driving Patients are advised to check how they feel when holding the steering wheel ‘If slippy, then not to drive wearing them’ (P05)
*Provision of written glove information* Most provided written glove information, either their own departments’ glove instructions sheet (*n* = 12) and/or the manufacturer’s instruction sheet (*n* = 9)

Key: *p* = Therapist Personal Identification Number.

Over half indicated they do not routinely evaluate the effectiveness of gloves in review appointments, primarily because they lack capacity. Often, identifying the effects of gloves had to be ad hoc using verbal feedback: ‘what finding them beneficial for’ (P15); and ‘have gloves helped with pain, stiffness, swelling, function and overall feeling of wellbeing?’ (P06) (Table 5). Therapists were asked about patient feedback they received during glove reviews:

• Benefits: reduced pain (*n* = 12); reduced swelling (*n* = 11); gloves are supportive (*n* = 9); activities are easier (*n* = 7); reduced morning stiffness (*n* = 6); warmth (*n* = 5); more acceptable than splints (*n* = 2); and can wear at work (*n* = 2).
• Problems: gloves too hot (*n* = 10); difficulty getting on and off (*n* = 7); wear out too quickly (*n* = 4); inconvenient as need to remove for wet activities/hygiene (*n* = 3); not liking pressure/tightness (*n* = 3); and no support (*n* = 3).

Therapists indicated arthritis gloves last for: day use only = 5.7 (SD 2.2) months; night use only = 7.9 (SD 2.8) months; day and night use = 4.5 (SD 1.9) months. It also ‘depends on how often the patient washes them. If vigilant about hygiene it may only be 2 months’ (P05). They will then need replacing.

### Annual estimated costs of arthritis glove provision

Most patients prescribed arthritis gloves are provided with a pair,^20^ around two-thirds continue wanting to wear them,^20,21^ and therapists indicated gloves need replacing every four to six  months and that they provide replacement gloves. From the regional survey data, based on estimated numbers of gloves provided per month in RA and HOA, NHS staff time (based on Band 6) taken to provide gloves, and average glove costs, we estimate the average cost to a rheumatology occupational therapy department of providing arthritis gloves annually (including providing one replacement pair; based on 2020 costs) is £14,631 (Supplementary File 5). There will be annual recurring costs if more than one replacement pair is issued, and patients require re-assessing to re-issue.

## Discussion

This survey was part of a programme of research about arthritis gloves, consisting of a systematic review,^16^ feasibility trial^20^ and this survey undertaken to assist planning^22^ and then conducting a randomised controlled trial.^21^ The survey clarified for the first time how arthritis gloves are being provided in clinical practice. Arthritis gloves are a popular intervention in RA but also now in HOA, with most responding rheumatology occupational therapists providing these, to about a third of the patients they see with RA and HOA. Clinical practice is more diverse than previously reported,^1,5,10,11^ as arthritis gloves are provided to a wider range of people with hand rheumatic and musculoskeletal conditions. Presence of hand symptoms and need for hand support are the main drivers for glove provision, rather than diagnosis. At the time of the survey, there was little published theorising how arthritis gloves work and no physiological studies of effects. Therapists’ comments about potential mechanisms of action, and how they explain this to patients, reflected what little theory was available. They also proposed mechanisms, such as compression providing support to improve hand function, to explain their use in the day-time. This use had not previously been researched.

Practice has changed since the 1990s. It is now more common to provide open-finger arthritis gloves, in contrast to only closed-finger gloves previously.^1–5^ Arthritis gloves are additionally used to improve day-time hand symptoms and hand function, previously only described in two case studies,^10,11^ rather than only for night-time relief of pain, swelling and stiffness. The main factors influencing day-time provision included moderate to severe persistent day-time hand pain, swelling and need for hand support for activities. Patient preference influences glove provision as these are considered as being better tolerated by some and considered more acceptable, particularly in early RA, than firmer day or night orthoses. If gloves are for night use only, closed-finger gloves may still be provided. The survey also identified therapists had clear contraindications for glove use.

Isotoner gloves tended to be more popular as considered a better made glove, providing greater compression, and more suitable for people with RA, who tend to have more severe hand swelling than in HOA. There was a preference for oedema gloves for HOA, as these are less compressive. However, glove cost was also a main driver for glove selection, as oedema gloves are around £3 cheaper per pair (at NHS cost) than Isotoner™ gloves.

Half of therapists used standardised assessments when deciding on glove provision, mostly focused on hand symptoms, particularly hand pain. The routine use of assessments to re-evaluate if gloves are effective was unusual, as constrained by lack of staff time.^23^ Therapists mainly relied on patient feedback with many commenting that this was largely positive. The commonest benefits reported were in reduced hand pain, swelling and improved hand function, reflecting the findings in our feasibility trial of patient-reported benefits.^20^ Key influences on glove provision in their therapy service were primarily other therapists’ feedback about glove benefits, information from courses and positive patient feedback. This reflected that, at the time of the survey, there was limited evidence from which to make clinical decisions, with conflicting findings about arthritis gloves’ effects^16^ and thus a reliance on clinical and patient opinion.

Previously, there was little published information available about how and when arthritis gloves should be worn, particularly in the day, or about contraindications for use. This survey identified clinical practice is to individualise wear regimens depending on patients’ symptom severity and functional needs. Therapists recommend patients wear gloves ‘as and when’ they need them during the day, and throughout the night if tolerated. Therapists also recommend that gloves should not be worn continuously in the day, but regularly removed to check for adverse effects, for hygiene reasons and hand exercises routinely performed on removal.

The findings of this survey contributed to planning a randomised controlled trial evaluating arthritis gloves, combined with findings from a feasibility trial, discussions with a panel of expert clinical therapists and patient representatives, as well as with the trial research team. We tested open-finger Isotoner^TM^ gloves in RA as the most popular glove and most common condition gloves are provided for. Key inclusion and exclusion criteria were based on factors influencing why therapists provide gloves and glove contraindications. Hand pain during activities in the day was the primary outcome as reducing this is a key aim for therapists providing gloves. Therapists anticipate two-thirds of patients will wear gloves long-term, with short-term use (i.e. up to 6 months) in early RA. This informed our primary end point of a 12-week follow-up, rather than one to 4 weeks in previous trials. Therapists’ recommendations for glove wear structured the wear regimens. Safety advice assisted standardising glove information provided verbally and in writing to trial participants. Information on glove appointment duration, hand assessments used and hand exercise provision influenced the trial treatment protocol. This helped ensure a pragmatic trial reflecting clinical practice was conducted.^21,22^

We subsequently completed the A-Gloves trial.^21^ When this survey was conducted (in 2015) therapists’ understanding of the mechanism of action, that is, pressure as the active ingredient of arthritis gloves, reflected the theoretical mechanisms described previously.^4,5^ Some also identified warmth, which is supported by previous research. Thermal pressure gradient arthritis gloves increased hand temperature by about 1°C, although so too did placebo non-stretch cotton gloves, with no differences in glove effects on hand symptoms apart from reduced finger joint swelling.^2^ Thermal pressure gradient arthritis gloves and placebo thermal gloves were also identified as having similar small effects on hand symptoms.^4^ The A-Gloves trial (*n* = 206) identified Isotoner™ gloves and placebo loose-fitting nylon oedema gloves (i.e. one to two sizes too big so not applying pressure) both had similar, but generally small and not clinically significant, effects on hand symptoms and hand function.^21^ Three-quarters of participants in both groups liked the gloves they were provided with and would continue to wear them, reporting that they liked the warmth provided with glove wear.^21,24^ The A-Gloves trial, and previous trials, suggest that any small benefits from glove wear are due to warmth rather than pressure.^2,4,21^ As warmth effects could be achieved by wearing ordinary open-fingered gloves, providing arthritis gloves can be questioned. However, if arthritis gloves continue in use, explanations to patients about warmth effects of gloves would reflect evidence now available.

The estimated annual arthritis glove costs for departments (for RA and HOA only) may be in the order of £4000, or £14,000 if staff costs for provision are included. This estimate included providing a replacement pair once, to about 70% of patients, reflecting clinical practice. Costs will be higher as gloves are provided to a wider range of people with hand rheumatological and musculoskeletal conditions and can be recurrently provided to a patient over years, for which re-assessment would likely be required. If patients buy their own replacement gloves, commercial prices vary from £20 to £32 per pair (depending on type and supplier) for the same types of gloves provided in the NHS. As gloves last four to 6 months, patients might pay £40–£90/year to continue arthritis glove wear. Given evidence that loose-fitting nylon or thermal gloves can have similar small effects to arthritis gloves, it would be cheaper for both therapy departments and patients, if patients are recommended to purchase ordinary open-finger, light-weight nylon (or similar) gloves for themselves.

The strengths of this study were that the questionnaire was developed with clinical therapists to capture what they considered key issues to investigate about arthritis glove provision. The limitations were that we did not have any patient and public involvement (PPI) partners assisting questionnaire design, as we had not yet recruited PPI members at this early stage of the research programme. The responses were from members of rheumatology occupational therapy groups and not all such therapists have joined those groups. Additionally, the detailed responses about clinical practice were from therapists in one region of England, and there is a possibility that practice could differ from the rest of the country as the North-West group members periodically exchange information about practice. However, most respondents were specialist therapists with many years of rheumatology experience, who indicated key influences on practice were from attending courses nationally as well as discussions with therapists regionally and nationally. There could have been some doubling of responses from North-West members answering both the regional and national surveys. Although informed they need not do so, we could not monitor if they did as the national survey was anonymous. A further limitation is that the estimated costs for glove provision are just that. There are many variables which can influence any one department’s costs.

## Conclusion

This survey identified that arthritis gloves are being used for a wider range of hand rheumatic and musculoskeletal conditions than previously reported. Use has extended to day-time as well as night-time wear. Key influences on practice have been clinical opinion and patient positive feedback, as evidence at the time of this survey was limited and conflicting in some respects. The A-Gloves trial, published since this survey, indicates that loose-fitting nylon gloves have similar effects to arthritis gloves, and the latter are not cost-effective. Consequently, it is important therapists review their clinical practice and departmental expenditure in arthritis glove provision. It could be that staff time is better spent providing hand exercises and/or ergonomics training (joint protection) using cognitive-behavioural approaches, which have both been found to be effective interventions in trials.^25,26^ Anecdotally, clinical practice is already changing to reduce provision of arthritis gloves. Conducting a similar online survey in the future will help to identify how practice changes in the light of evolving research.

## Supplemental Material

sj-pdf-1-hth-10.1177_17589983211060620 – Supplemental Material for Arthritis glove provision in rheumatoid arthritis and hand osteoarthritis: A survey of United Kingdom rheumatology occupational therapists Click here for additional data file. Supplemental Material, sj-pdf-1-hth-10.1177_17589983211060620 for Arthritis glove provision in rheumatoid arthritis and hand osteoarthritis: A survey of United Kingdom rheumatology occupational therapists by Alison Hammond and Yeliz Prior in Hand Therapy

sj-pdf-2-hth-10.1177_17589983211060620 – Supplemental Material for Arthritis glove provision in rheumatoid arthritis and hand osteoarthritis: A survey of United Kingdom rheumatology occupational therapists Click here for additional data file. Supplemental Material, sj-pdf-2-hth-10.1177_17589983211060620 for Arthritis glove provision in rheumatoid arthritis and hand osteoarthritis: A survey of United Kingdom rheumatology occupational therapists by Alison Hammond and Yeliz Prior in Hand Therapy

sj-pdf-3-hth-10.1177_17589983211060620 – Supplemental Material for Arthritis glove provision in rheumatoid arthritis and hand osteoarthritis: A survey of United Kingdom rheumatology occupational therapists Click here for additional data file. Supplemental Material, sj-pdf-3-hth-10.1177_17589983211060620 for Arthritis glove provision in rheumatoid arthritis and hand osteoarthritis: A survey of United Kingdom rheumatology occupational therapists by Alison Hammond and Yeliz Prior in Hand Therapy

sj-pdf-4-hth-10.1177_17589983211060620 – Supplemental Material for Arthritis glove provision in rheumatoid arthritis and hand osteoarthritis: A survey of United Kingdom rheumatology occupational therapists Click here for additional data file. Supplemental Material, sj-pdf-4-hth-10.1177_17589983211060620 for Arthritis glove provision in rheumatoid arthritis and hand osteoarthritis: A survey of United Kingdom rheumatology occupational therapists by Alison Hammond and Yeliz Prior in Hand Therapy

sj-pdf-5-hth-10.1177_17589983211060620 – Supplemental Material for Arthritis glove provision in rheumatoid arthritis and hand osteoarthritis: A survey of United Kingdom rheumatology occupational therapists Click here for additional data file. Supplemental Material, sj-pdf-5-hth-10.1177_17589983211060620 for Arthritis glove provision in rheumatoid arthritis and hand osteoarthritis: A survey of United Kingdom rheumatology occupational therapists by Alison Hammond and Yeliz Prior in Hand Therapy

## Online supplementary files

Supp File I Arthritis glove survey: figures 1 and 2

Supp File II Arthritis glove national survey instrument

Supp File III Arthritis glove survey: factors influencing decisions to provide gloves in RA and HOA

Supp File IV Arthritis glove survey: Therapists reasons for providing Isotoner and/or oedema gloves

Supp File V Arthritis glove survey: Estimated costs of glove provision in RA and HOA
