# Supplementary material for: Arthritis glove provision in rheumatoid arthritis and hand osteoarthritis: A survey of United Kingdom rheumatology occupational therapists
Source: Hand Ther. 2022 Jan 5;27(1):3–13. doi: 10.1177/17589983211060620 (PMC10584060; doi:10.1177/17589983211060620)
Supplement: sj-pdf-1-hth-10.1177_17589983211060620 – Supplemental Material for Arthritis glove provision in rheumatoid arthritis and hand osteoarthritis: A survey of United Kingdom rheumatology occupational therapists [file sj-pdf-1-hth-10.1177_17589983211060620.pdf]

Supplementary File I: Figure 1 Isotoner glove and Figure 2 Oedema arthritis glove

Figure 1: Isotoner™ arthritis glove

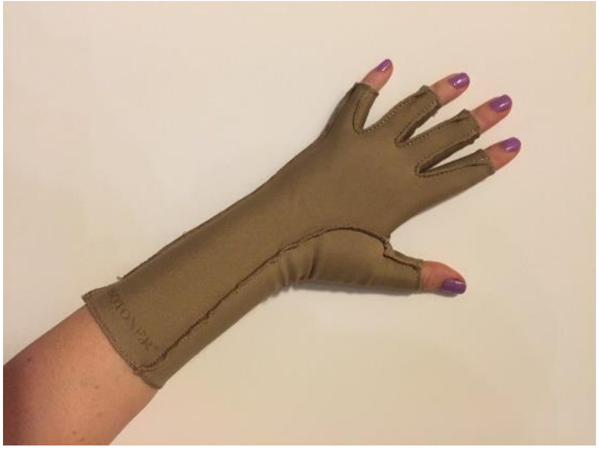

Figure 2: Oedema arthritis glove

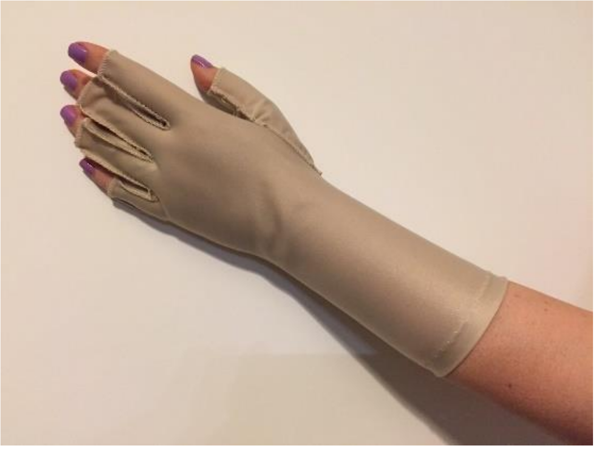

Hammond A, Prior Y. Arthritis glove provision in rheumatoid arthritis and hand osteoarthritis: a survey of United Kingdom rheumatology occupational therapists. Hand Therapy 2021
