# Supplementary material for: Arthritis glove provision in rheumatoid arthritis and hand osteoarthritis: A survey of United Kingdom rheumatology occupational therapists
Source: Hand Ther. 2022 Jan 5;27(1):3–13. doi: 10.1177/17589983211060620 (PMC10584060; doi:10.1177/17589983211060620)
Supplement: sj-pdf-2-hth-10.1177_17589983211060620 – Supplemental Material for Arthritis glove provision in rheumatoid arthritis and hand osteoarthritis: A survey of United Kingdom rheumatology occupational therapists [file sj-pdf-2-hth-10.1177_17589983211060620.pdf]

Supplementary File II: The 12 questions in the arthritis glove national rheumatology occupational therapists' survey.

1. How many years rheumatology therapy experience do you have?
2. What NHS Band are you?
3. What type of hospital do you work in? [community hospital; district general hospital; university hospital; other, please state)4 Do you provide compression gloves for RA? (Yes; No)
4. a. If yes, how many years of experience do you have in providing compression gloves in arthritis? (less than 1 year; between 1 to 2 years; between 2 to 3 years; between 3 to 5 years; more than 5 years).
4. b. If you do not provide compression gloves in RA, why is this (no clinical experience of providing gloves; no evidence of their effectiveness available; no budget to provide gloves; I do not consider gloves are effective; I consider other splints (e.g. resting splints) a better option; other please state).
5. Do you provide compression gloves in …options: early RA; established RA; other arthritis conditions, please state).
6. In early RA do you provide compression gloves: prior to DMARDs, when stable on DMARDs; other, please state)
7. Approximately how many people with RA does your OT department treat each month?
8. To how many patients with RA does your OT department issue compression gloves to per month?
9. Which types of compression gloves do you provide? (Isotoner; oedema; own manufactured, other, please state).
10. Do you provide: three-quarter length finger gloves; full finger length gloves.
11. When do you recommend people with RA wear compression gloves? Options: day, night; during a flare
12. a. Do you provide replacement gloves when the first gloves issued wear out? (Yes/No)
12. b. Do you inform patients about glove manufacturers' / suppliers contact details in order to purchase replacement gloves themselves? (Yes/No)
12. c. Do you charge for subsequent provision of gloves? (Yes/No)

An open text box was provided inviting respondents to make any other comments about glove provision.

Hammond A, Prior Y. Arthritis glove provision in rheumatoid arthritis and hand osteoarthritis: a survey of United Kingdom rheumatology occupational therapists. Hand Therapy 2021
