# Supplementary material for: Arthritis glove provision in rheumatoid arthritis and hand osteoarthritis: A survey of United Kingdom rheumatology occupational therapists
Source: Hand Ther. 2022 Jan 5;27(1):3–13. doi: 10.1177/17589983211060620 (PMC10584060; doi:10.1177/17589983211060620)
Supplement: sj-pdf-3-hth-10.1177_17589983211060620 – Supplemental Material for Arthritis glove provision in rheumatoid arthritis and hand osteoarthritis: A survey of United Kingdom rheumatology occupational therapists [file sj-pdf-3-hth-10.1177_17589983211060620.pdf]

Supplementary File III: Factors influencing therapists' decision to provide gloves in in early and established rheumatoid arthritis and in hand osteoarthritis (median; interquartile range: North-West region survey (n=17).

**Factors:**	**Early RA (< 2 years)**	**Established RA (>2 years)**	**HOA**
High levels of day pain	5 (5-5)	5 (5-5)	5 (5-5)
High levels of night pain	5 (4-5)	5 (3.5 – 5)	5 (4-5)
Hand joint swelling	5 (4-5)	5 (4-5)	5 (3-5)
Patient tolerates gloves better than resting (firmer) splints at night	5 (4-5)	5 (4-5)	5 (4-5)
At patient request as had gloves before	5 (4-5)	5 (4-5)	5 (4-5)
Patient prefers gloves to wrist (other) splints in day	5 (4-5)	5 (4-5)	4 (3-5)
Gloves easier to manage than resting splints if patient gets up frequently at night	5 (3-5)	5 (3-5)	5 (3.5-5)
Provide hand support during day	4 (4-5)	5 (4-5)	5 (4-5)
Sleep disturbance due to hand pain	4 (3.5-5)	5 (4-5)	5 (4-5)
Early morning stiffness reducing hand function in morning	4 (3-5)	4 (3-5)	4 (2.5-4)
Gloves more acceptable in early RA	4 (2-5)	-	-

**Factors:**	**Early RA (< 2 years)**	**Established RA (>2 years)**	**HOA**

Limited hand movement	3 (3-4)	3 (3-4)	3 (2.5-4)
Maintain a comfortable position at night	3 (2-4)	3 (2-5)	3 (1.3-4.5)
Reduce clawing/strong finger flexion at night	1 (0-3)	1 (0-3)	2 (0-3.5)

Key: RA = rheumatoid arthritis; HOA = hand osteoarthritis; Scale: 0 = not important/relevant; 1 = low importance; 5 = high importance. Items in italics are patient preferences.

Hammond A, Prior Y. Arthritis glove provision in rheumatoid arthritis and hand osteoarthritis: a survey of United Kingdom rheumatology occupational therapists. Hand Therapy 2021
