# Supplementary material for: Arthritis glove provision in rheumatoid arthritis and hand osteoarthritis: A survey of United Kingdom rheumatology occupational therapists
Source: Hand Ther. 2022 Jan 5;27(1):3–13. doi: 10.1177/17589983211060620 (PMC10584060; doi:10.1177/17589983211060620)
Supplement: sj-pdf-4-hth-10.1177_17589983211060620 – Supplemental Material for Arthritis glove provision in rheumatoid arthritis and hand osteoarthritis: A survey of United Kingdom rheumatology occupational therapists [file sj-pdf-4-hth-10.1177_17589983211060620.pdf]

Supplementary File IV: Therapists' reasons for providing Isotoner, oedema or bespoke gloves; and glove modifications made: North-West region survey (n=17).

Isotoner gloves:

Better quality and fit: "superior product, more robust" (P13); a wider wrist opening allowing easier fitting" than oedema gloves (P03, P06)

Better for those with greater hand symptoms: "give more support and compression than oedema gloves" (P06, P14); "tend to use more with RA patients due to symptoms – more swollen joints/and swelling, pain and stiffness than HOA patients" (P03); for patients "with significant swelling or need more support than provided by oedema gloves" (P21).

Oedema gloves:

Less compression: "not as tight" as Isotoner gloves (P01,P04)

Cost: "cost implications" (P03: oedema gloves are cheaper)

Size: "if Isotoners are too big" (P06: oedema gloves are available in extra small)

Preferred for HOA: "tend to provide for OA patients as less compression [and they are] provided for other reasons, e.g. warmth to improve mobility…less support but more flexible for functional activities and patients tend to find Isotoners too strong" (P03).

Bespoke gloves:

Size: "when a smaller or larger glove than commercially available is needed" (P05)

Deformity: "too much deformity to fit a commercial glove" (P09)

Don/doff: "Unable to don and doff a commercial glove, so bespoke gloves are made with a zip" (P18, P25).

Glove modifications:

- shortening fingers (P02,P09); removing finger/s e.g. due to severe flexion contractures (P02); taking in seams to improve fit (P02, P18); releasing finger stitching if too tight

(P08); making slits at the wrist in oedema gloves to make donning/doffing easier (P15); and adding a Lycra insert at the wrist if the wrist/forearm are very broad (P18).

Key: P = therapist's Personal Identification Number

Hammond A, Prior Y. Arthritis glove provision in rheumatoid arthritis and hand osteoarthritis: a survey of United Kingdom rheumatology occupational therapists. Hand Therapy 2021
