# Supplementary material for: Arthritis glove provision in rheumatoid arthritis and hand osteoarthritis: A survey of United Kingdom rheumatology occupational therapists
Source: Hand Ther. 2022 Jan 5;27(1):3–13. doi: 10.1177/17589983211060620 (PMC10584060; doi:10.1177/17589983211060620)
Supplement: sj-pdf-5-hth-10.1177_17589983211060620 – Supplemental Material for Arthritis glove provision in rheumatoid arthritis and hand osteoarthritis: A survey of United Kingdom rheumatology occupational therapists [file sj-pdf-5-hth-10.1177_17589983211060620.pdf]

Supplementary File V: Estimated costs of glove provision for rheumatoid arthritis and hand osteoarthritis, based on averages from North-West region survey (n=17).

	**Glove numbers and NHS costs:**<br><br>**staff time and costs**	**Annual Cost**
**No. gloves (pairs) provided in RA and HOA:**		
- per month	13 + 7 = 20	
- per year	240	
**Average NHS glove costs:**		
- Isotoner™ (per pair)	£11.87 (range £10 - £13.75)	
- Oedema (per pair)	£8.50 (range £7-£10)	
- Average cost of Isotoner or oedema glove provision	£10.18	
**Average glove cost per year:**		
Provision of first glove pair	240 x £10.18	£2,443
**Replacement gloves:**		
Approx. 70% require replacement gloves after 6 months	168 x £10.18	£1,710
**Estimated annual glove costs:**		
(one pair replacement gloves only provided)		£4,153
**NHS staff costs**:		
Band 6 therapist costs = £50/hour @52.4 minutes to provide gloves	£43.66	
Annual staff costs*	240 x £43.66	£10,478
**Estimated total (gloves plus staff costs)**		**£14,631**

Key: * = no additional staff time for providing one replacement pair included.

Hammond A, Prior Y. Arthritis glove provision in rheumatoid arthritis and hand osteoarthritis: a survey of United Kingdom rheumatology occupational therapists. Hand Therapy 2021
